# Discordance in Retinal and Choroidal Vascular Densities in Patients with Type 2 Diabetes Mellitus on Optical Coherence Tomography Angiography

**DOI:** 10.1155/2021/8871602

**Published:** 2021-02-09

**Authors:** Ho Ra, Nam Yeo Kang, Jiyun Song, Junhyuck Lee, Inkee Kim, Jiwon Baek

**Affiliations:** Department of Ophthalmology, Bucheon St. Mary's Hospital, College of Medicine, The Catholic University of Korea, Seoul, Republic of Korea

## Abstract

**Purpose:**

In the present study, the retinal and choroidal vascular densities (VDs) in type 2 diabetes mellitus (DM) patients were analyzed using optical coherence tomography angiography (OCTA).

**Methods:**

The study included 282 eyes of 152 patients with type 2 DM (114 without retinopathy, 79 nonproliferative diabetic retinopathy (NPDR), 48 severe NPDR, and 41 proliferative diabetic retinopathy (PDR) eyes). The superficial and deep retinal vessel, choriocapillaris, and choroidal VDs were measured using a binarization method on OCTA images. VDs were compared based on retinopathy severity. Correlations among densities were analyzed.

**Results:**

Retinal and choriocapillaris VDs were lower in PDR than in NPDR (all *P* < 0.05). Correlation analysis showed significant positive correlations among densities of superficial and deep retinal vessels and choriocapillaris (all *P* < 0.001). Choroidal VD showed a negative correlation with superficial and deep retinal vessels and choriocapillaris (all *P* < 0.001). Retinal and choriocapillaris VDs showed a negative correlation with diabetic retinopathy (DR) grade (all *P* < 0.001); however, the choroidal VD showed a weak positive correlation (*P*=0.030).

**Conclusion:**

Choroidal VD increased as retinal and choriocapillaris VDs decreased, indicating that the outer layer of the choroid is less affected by DR severity and VD of larger choroidal vessels may even be increased as a compensatory mechanism for decreased retinal and choriocapillaris VDs in type 2 DM patients.

## 1. Introduction

Diabetic retinopathy (DR) is a major cause of blindness in adults worldwide [1]. Although the pathogenic mechanism of DR is not fully understood due to its complexity, upregulation of angiogenic cytokines and inflammatory mediators caused by metabolic disturbances is considered a core mechanism resulting in a chain of pathological processes in DR [2]. Retinal vessels are influenced by a hypoxic state which is caused by chronic hyperglycemic status in DR eyes. Hypoxia-driven angiogenic factors, including vascular endothelial growth factor (VEGF), cause vasodilation and leakage of retinal capillaries and may eventually lead to proliferation of new vessels [3].

Vascular involvement in DR pathogenesis has previously been studied. In recent years, the introduction of optical coherence tomography angiography (OCTA) has facilitated investigations on retinal vessels as well as choriocapillaris and large choroidal vessels. An important finding in vasculature changes in DR using OCTA includes enlargement of the foveal avascular zone [4]. In addition, lower retinal vascular density (VD) in DR eyes has consistently been reported in previous studies [5–7]. Furthermore, retinal VD was shown associated with disease severity or visual function [6].

Although vascular features of the retina in diabetes mellitus (DM) patients are similar to the above-described changes, the VD change of retina and choroid and their direct correlations have not yet been clarified. In the current study, superficial and deep retinal vessel, choriocapillaris, and choroidal vessel densities in type 2 DM patients were quantitatively analyzed using OCTA and compared based on retinopathy severity. In addition, the correlation among densities was investigated.

## 2. Materials and Methods

This retrospective cross-sectional case series study was performed in the Department of Ophthalmology at Bucheon St. Mary's Hospital, The Catholic University of Korea (Gyeonggi-do, Republic of Korea). The study was approved by the hospital's institutional review board and conducted according to the Declaration of Helsinki. Informed consent was waived due to the retrospective nature of the study.

### 2.1. Patients

The study group consisted of consecutive type 2 DM patients who visited the ophthalmology outpatient department at Bucheon St. Mary's Hospital between December 2019 and February 2020. All patients included in the study were diagnosed with type 2 DM by doctors in the endocrinology department of the hospital. Type 2 DM was diagnosed if the patient exhibited a fasting plasma glucose level of ≥126 mg/dl or a 2 h postglucose level of ≥200 mg/dl after a 75 g oral glucose tolerance test [8]. The severity of DR was evaluated in accordance with the Early Treatment Diabetic Retinopathy Study standard grading protocols by two retinal specialists with an experience in DR over 10 years (H.R. and J.B.) [9].

Each patient underwent a complete ophthalmologic examination, including measurement of best-corrected visual acuity (BCVA), slit-lamp examination, and dilated fundus examination using mydriatic ultra-widefield color fundus photography (Optos California P200DTx icg; Optos, Dunfermline, United Kingdom) and Heidelberg HRA2 Spectralis OCTA device (Heidelberg Engineering®, Germany). DR grade was determined based on the modified Early Treatment Diabetic Retinopathy Study grade [9].

Demographic and clinical data including age, sex, coexistence of hypertension, duration of DM, systolic and diastolic blood pressures, serum levels of random glucose, and hemoglobin A1c (HbA1c) were collected.

Exclusion criteria were as follows: (1) low-quality images due to significant cataract, corneal opacities, vitreous hemorrhages, or poor cooperation and images with Q score lower than 30; (2) eyes with diabetic macular edema (i.e., central macular thickness (CMT) of 350 *μ*m or more and/or eyes with intra- or subretinal fluids) to eliminate interference of macular fluid on the vessel density analysis; (3) receiving any prior treatment for DR including anti-VEGF therapy, intraocular or periocular steroid, laser photocoagulation, or vitrectomy; and (4) presence of any significant retinal pathology other than PDR (including pathologic myopia, retinal vessel occlusion, other vasculitis symptoms, or retinal detachment).

### 2.2. Image Analysis

**Figure 1:**
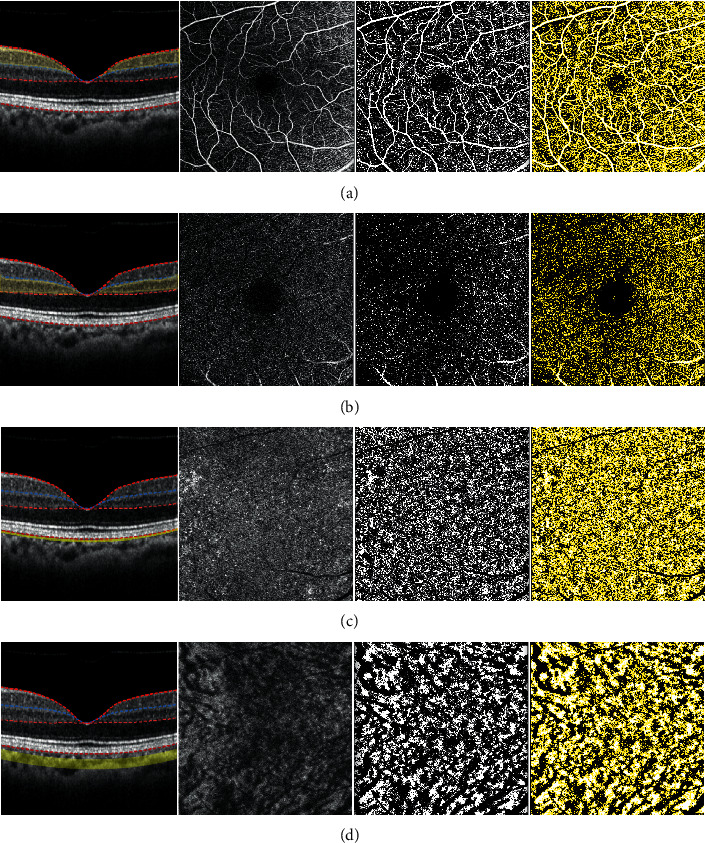
Vessel density measurements of each layer. En face 3 × 3 mm2 optical coherence tomography angiography (OCTA) images of (a) superficial vascular complex (SVC), (b) deep vascular complex (DVC), (c) choriocapillaris, and (d) choroid were analyzed. Each slab was automatically obtained using predefined settings of the Spectralis OCTA system. SVC consisted of nerve fiber layer vascular plexus and superficial vascular plexus, and DVC consisted of intermediate capillary plexus and deep capillary plexus. Choriocapillaris slab is generated by segmentation of 10 to 30 *μ*m below Bruch's membrane and choroid is defined as choroidal areas below the choriocapillaris up to chorioscleral junction (first and second column). Image binarization was performed using Niblack's method with a 30° radius (third column). The white region was considered the vascular area in SVC, DVC, and choriocapillaris slabs and the black region was considered the vascular area in choroid slab (forth column). The number of pixels was obtained, and flow density was calculated by dividing the number of pixels of the vascular area by the total region of interest.

*En face* 3 × 3 mm^2^ macular area OCTA images of superficial vascular complex (SVC), deep vascular complex (DVC), choriocapillaris, and choroid, centered at the fovea were obtained. Each slab was automatically obtained using predefined settings of Spectralis OCTA system. SVC consisted of nerve fiber layer vascular plexus and superficial vascular plexus, and DVC consisted of intermediate capillary plexus and deep capillary plexus. Choriocapillaris slab is generated by segmentation of 10 to 30 *μ*m below Bruch's membrane and choroid is defined as choroidal areas below the choriocapillaris up to chorioscleral junction. Image binarization was performed using Niblack's method with a 30° radius using FIJI software (an expanded version of ImageJ version 1.51a, available at fiji.sc, free of charge). The white region was considered the vascular area in SVC, DVC, and choriocapillaris slabs and the black region was considered vascular area in choroid slab. The number of pixels was obtained and flow density was calculated by dividing the number of pixels of the vascular area by the total region of interest (Figure 1). Choroidal VD on OCTA was compared and validated with choroidal VD measured on the structural *en face* image.

### 2.3. Statistical Analysis

Statistical analysis was performed with SPSS for Windows (version 23.0.1; SPSS Inc., Chicago, IL, USA). For statistical analysis, the Snellen BCVA was converted to a logarithm of the minimal angle of resolution (logMAR). Values of continuous variables are presented as means ± standard deviation. One-way analysis of variance (ANOVA) was used to compare continuous variables among and between groups. Mann-Whitney and Kruskal-Wallis tests were used when a normal distribution could not be confirmed. Post hoc analysis was conducted using the Bonferroni test. Categorical variables between groups were compared using the chi-square test. Standardized adjustment was used as the post hoc test after the chi-square test. Pearson's correlation analysis was used to determine the coefficients of correlation between each VD and VDs and clinical parameters following confirmation of normal distribution. Spearman's correlation was used when normal distribution was not confirmed. Multivariate linear regression analysis was done for relevant variables. A *P* value < 0.05 was considered statistically significant.

## 3. Results

### 3.1. Demographic and Clinical Features

Of 295 consecutive eyes, we excluded 7 eyes due to poor image quality and 6 eyes due to concomitant macular diseases. In total, the present study included 282 eyes of 152 type 2 DM patients. Among the 282 eyes, 114 eyes did not show retinopathy, 79 had nonproliferative diabetic retinopathy (NPDR), 48 eyes had severe NPDR, and 41 eyes had proliferative diabetic retinopathy (PDR). All patients were Korean. The mean age was 60.12 ± 12.17 years, 57% were male, and 57% and 13% had comorbid hypertension (HTN) and chronic kidney disease (CKD), respectively. The mean duration of DM was 9.67 ± 7.91 years. The mean BCVA was 0.13 ± 0.29 logMAR. Age, sex distribution, DM duration, comorbid HTN and CKD, BCVA, random serum glucose level, and CMT did not differ significantly between DR grades (all *P* ≥ 0.084). DM duration was significantly shorter in patients without DR compared with other grades (all *P* < 0.001) and longer in patients with PDR than without DR or NPDR (*P* < 0.001 and *P*=0.034, respectively). Baseline demographic and clinical features of the study eyes are summarized in Table 1.

### 3.2. Comparing Vessel Densities of Different Retinal and Choroidal Layers among DR Grades

**Figure 2:**
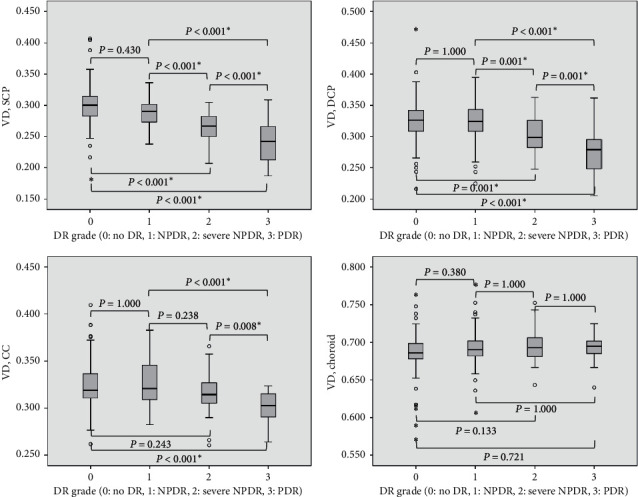
Comparison of vessel densities and grades of diabetic retinopathy (DR). Vascular densities (VDs) of superficial vascular complex (SVC), deep vascular complex (DVP), and choriocapillaris differed based on DR grades (all *P* < 0.001). Post hoc analysis showed that severe nonproliferative diabetic retinopathy (NPDR) and proliferative diabetic retinopathy (PDR) eyes had lower VDs of SVC and DVC compared with their previous grades (all *P* < 0.001). PDR eyes also showed lower choriocapillaris VD compared with lower DR grades (all *P* < 0.008).^*∗*^Statistically significant *P* value.

The mean VD of each layer was 0.28 ± 0.03, 0.31 ± 0.04, 0.32 ± 0.02, and 0.69 ± 0.02 for SVC, DVC, choriocapillaris, and choroidal vessels, respectively. It was 0.69 ± 0.2 for choroidal vessel measured in structural *en face* image. The choroidal VDs measured in OCTA and structural *en face* showed a strong positive correlation with a high intraclass correlation coefficient of 0.852 (*r* = 0.743; *P* < 0.001). The ANOVA analysis showed that VDs of SVC, DVC, and choriocapillaris differed based on DR grade (all *P* < 0.001). Post hoc analysis showed that severe NPDR and PDR eyes had lower VDs of SVC and DVC compared with their previous grades (all *P* < 0.001, Figure 2). PDR eyes also showed lower choriocapillaris VD compared with lower DR grades (all *P* < 0.008).

### 3.3. Correlation between Vessel Densities and Clinical Parameters

**Figure 3:**
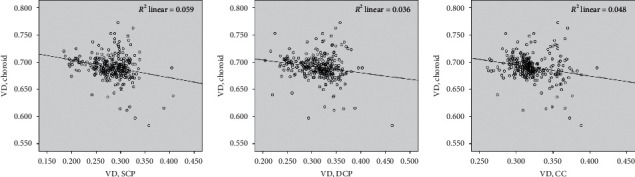
Correlation between choroidal vascular densities (VDs) and VDs of other layers. Choroidal vascular density (VD) showed weak negative correlations with VDs of other layers (*r* = −0.243, −0.189, and −0.220; *P* < 0.001, = 0.001, and < 0.001, for superficial capillary complex (SVC), deep capillary complex (DVC), and choriocapillaris, respectively).

Vessel densities of SVC, DVC, and choriocapillaris were negatively correlated with DR grade (*r* = -0.573, −0.441, and −0.309, respectively; all *P* < 0.001; Figure 3); however, choroidal VD was positively correlated with DR grade (*r* = 0.129; *P*=0.030). Age was negatively correlated with VD of SVC (*r* = −0.120; *P*=0.043). Vessel densities of SVC and DVC were negatively correlated with DM duration and comorbid HBP (*r* = −0.213 and −0.165; *P* < 0.001 and  = 0.005 for SVC, respectively, and *r* = −0.142 and −0.148; *P*=0.017 and = 0.013 for DVC, respectively). The correlation between VDs and clinical parameters is summarized in Table 2. Multivariate regression analysis including DR grade, age, DM duration, and HTN showed that DR grade was an independent factor associated with VDs of SVC and DVC (*B* = −0.019 and −0.015; *P* < 0.001 and < 0.001, resp.). Age correlated with DM duration (*r* = 0.343; *P* < 0.001) and DM duration correlated with DR grades (*r* = 0.390; *P* < 0.001).

### 3.4. Correlation between Vessel Densities of Different Retinal and Choroidal Layers

Vessel densities of SVC, DVC, and choriocapillaris were strongly positively correlated (all *P* < 0.001, Table 3). Negative correlations were observed between choroidal VD and VDs of other layers (*r* = −0.243, −0.189, and −0.220; *P* < 0.001, = 0.001, and < 0.001, for SVC, DVC, and choriocapillaris, respectively).

## 4. Discussion

Measuring VDs using OCTA is useful for investigating the pathophysiology of retinal disease including DR in which vascular changes are the main process of the disease. In the present study, VDs of SVC, DVC, choriocapillaris, and choroid were analyzed in type 2 DM patients. The results showed a significant negative correlation between VDs of retinal layers with DR grades. However, VDs of larger choroidal vessels showed different association with the DR grades. The association between each VD of retinal layers and larger choroidal also revealed some differences.

The VDs of SVC, DVC, and choriocapillaris were significantly decreased as the severity of DR increased. Age and DM duration were also associated with VD which is in agreement with findings from previous studies. Retinal VDs were associated with DR severity, older age, higher HbA1c level, and the presence of DME in previous studies [7, 10]. In the present study, eyes with DME were excluded because DME can affect VDs as well as causing inaccurate measurement of VD. Age strongly correlated with DM duration and DM duration strongly correlated with DR grades; therefore, multivariate analysis showed that only DR severity was an independent factor associated with VDs of SVC and DVC. Definitive correlation was not observed between VDs and HbA1c level in the present study. This discordance might have been caused by different medical management strategies for DM. Further prospective controlled studies are warranted to clarify the correlation between HbA1c and VDs.

A significant association of DR with retinal, SVC, and DVC VDs has been reported in many previous studies and decreases in vessel densities in DR have been suggested [7, 11, 12]. Because the retina has nonanastomotic end arteries, the pathophysiology of DM affects retinal vessels [13, 14]. However, choroidal vessels can also be affected by DR status. Choriocapillaris perfusion was decreased in DM patients even without retinopathy in studies using OCTA, indicating that decreased choriocapillaris perfusion may exist before clinically detectable DR [7, 12, 15, 16]. In addition, the decrease in choriocapillaris VD was correlated with DR severity [7]. In the current study, choriocapillaris VD also decreased with increasing severity of DR. This finding is supported by other previous histopathological study results showing that a decrease in the alkaline phosphatase enzyme activity is associated with choriocapillaris loss in DM eyes [17, 18].

Conversely, choroidal VD did not significantly differ among various DR grades and even had a positive correlation with DR grades based on correlation analysis. The choroidal VD also negatively correlated with VDs of SVC, DVC, and choriocapillaris; however, other layers were positively correlated. Choroidal vascularity index (CVI) had been calculated in DR eyes and decreased CVI in PDR eyes was reported [19, 20]. In the current study, choriocapillaris VD was decreased with increased severity of DR, similar to other studies; however, choroidal VD was not decreased. Measurement of CVI includes all layers of the choroid including choriocapillaris as well as Sattler's and Haller's layers. The feasibility of layer-by-layer analysis is a distinctive advantage of OCTA including analysis for choroidal sublayer [21, 22]. The choroid slab used in the present study included choroidal area under the choriocapillaris and, therefore, represented the larger choroidal vessel status. In addition, CVI measurements are usually performed in one B-scan image which crosses the fovea. The *en face* image of OCTA can represent the general status of the larger choroidal vessel at a 3 × 3 mm2 macular area. The main physiological function of the choroid is to provide oxygen and nutrients to the highly metabolic outer retinal layers [23]. The positive correlation of choroidal VD with DR grade may suggest a compensatory mechanism of deep choroidal vessels in response to hypoxic status and hypoxic damage occurs in the retina. Alternatively, dilation of choroidal venule could be a reactive change to the occlusion choriocapillaris, as a shunt in retinal capillary occlusion may lead to venous beading in the diabetic retina [24].

The present study had several limitations. First, due to the retrospective design, factors that could affect clinical parameters, such as HbA1c and glucose levels, could not be controlled. Second, selection bias may have existed due to the exclusion of eyes with DME; however, we believe that disadvantages from excluding DME are outweighed by improved clarity of analysis. Third, the sample size of the current study was not large. Research with larger sample sizes is required to validate the results of this study. In addition, this study did not utilize an additional method for projection artifact reduction or not analyze the impact of signal strength, which can cause errors in VD analysis. Nonetheless, we minimized the error by including images with high Q score and excluding images with considerable artifacts. Also, the Spectralis OCTA system itself provides projection artifact removal. To the best of our knowledge, the association between choroidal vessel and DR grades or direct correlation among densities of retinal and choroidal vessels has not yet been reported in any study. The correlation between clinical parameters, as well as retinal and choroidal VDs, is a strength of the current study.

In conclusion, choroidal VD increased as retinal and choriocapillaris VDs decreased, indicating that the choroidal large vessel layer is less affected by DR severity, and larger choroidal vessel VD may even be increased as a compensatory mechanism for decreased retinal and choriocapillaris VDs in type 2 DM patients. However, interpretation of the results should be made with caution because the correlation was not strong. We believe that the results of this study can improve the understanding of the hemodynamics associated with DR.

## Figures and Tables

**Table 1:** Clinical characteristics of study eyes.

Variables	Total eyes	No DR	NPDR	Severe NPDR	PDR	*P* value
*N*	282	114	79	48	41	
Age (years), mean ± SD	60.12 ± 12.17	59.16 ± 12.82	61.51 ± 11.47	59.31 ± 12.19	61.05 ± 11.73	0.537
Sex (male, *n*) (%)	160 (57)	152 (54)	166 (59)	168 (60)	152 (54)	0.778
DM duration (years)	9.67 ± 7.91	6.12 ± 6.33	10.59 ± 7.34	12.42 ± 6.92	14.51 ± 9.73	>0.001^*∗*^
HTN (*n*) (%)	160 (57)	147 (52)	178 (63)	149 (53)	200 (71)	0.084
CKD (*n*) (%)	37 (13)	28 (10)	54 (19)	42 (15)	20 (7)	0.175
BCVA (LogMAR), mean ± SD	0.13 ± 0.29	0.15 ± 0.33	0.09 ± 0.21	0.12 ± 0.28	0.18 ± 0.33	0.436
HbA1c (%), mean ± SD	7.19 ± 1.43	6.84 ± 1.37	7.49 ± 1.5	7.34 ± 1.41	7.39 ± 1.35	0.009^*∗*^
Glucose (mg/dL), mean ± SD	152.67 ± 60.66	147.13 ± 46.25	157.11 ± 71.28	156.06 ± 64.03	155.51 ± 70.23	0.658
CMT (um), mean ± SD	264.41 ± 43.24	262.96 ± 39.85	268.27 ± 42.2	268.35 ± 44.46	256.41 ± 52.3	0.467

DR: diabetic retinopathy; NPDR: nonproliferative diabetic retinopathy; PDR: proliferative diabetic retinopathy; SD: standard deviation; DM: diabetes mellitus; HTN: hypertension; CKD: chronic kidney disease; BCVA: best-corrected visual acuity; HbA1c: hemoglobin A1c; CMT: central macular thickness.^*∗*^Statistically significant value.^*∗*^P value for DM duration should be < 0.001.

**Table 2:** Correlation between vessel densities and clinical parameters.

Layers		DR grade	Age	Sex	DM duration	HTN	CKD	BCVA	HbA1c	Glucose	CMT
VD; SVC	Pearson correlation	−0.573^*∗*^	−0.120^*∗*^	0.075	−0.213^*∗*^	−0.165^*∗*^	0.023	−0.0092	0.047	0.115	0.094
	Sig. (2-tailed)	<0.001	0.043	0.212	<0.001	0.005	0.696	0.126	0.436	0.054	0.115
VD; DVC	Pearson correlation	−0.441^*∗*^	−0.013	0.073	−0.142^*∗*^	−0.148^*∗*^	0.002	−0.059	0.028	0.078	0.066
	Sig. (2-tailed)	<0.001	0.831	0.224	0.017	0.013	0.967	0.323	0.640	0.193	0.267
VD; choriocapillaris	Pearson correlation	−0.309^*∗*^	0.104	0.010	−0.069	−0.035	0.047	−0.022	0.102	0.057	0.022
	Sig. (2-tailed)	<0.001	0.081	0.861	0.248	0.554	0.431	0.715	0.088	0.343	0.715
VD; choroid	Pearson correlation	0.129^*∗*^	−0.008	−0.089	0.031	0.063	0.060	0.005	0.032	0.063	−0.017
	Sig. (2-tailed)	0.030	0.892	0.137	0.605	0.288	0.319	0.937	0.589	0.290	0.777

DR: diabetic retinopathy; DM: diabetes mellitus; HTN: hypertension; CKD: chronic kidney disease; BCVA: best-corrected visual acuity; HbA1c: hemoglobin A1c; CMT: central macular thickness; VD: vessel density; SVC: superficial vascular complex; DVP: deep vascular complex; CC: choriocapillaris. ^*∗*^Statistically significant value.

**Table 3:** Correlation between vessel densities of different retinal and choroidal layers.

Layers		VD; SVC	VD; DVC	VD; CC	VD; choroid
VD; SCP	Pearson correlation^*∗*^	1	0.714	0.439	−0.243
	Sig. (2-tailed)		0.000	0.000	0.000
VD; DVC	Pearson correlation^*∗*^	0.714	1	0.611	−0.189
	Sig. (2-tailed)	0.000		0.000	0.001
VD; CC	Pearson correlation^*∗*^	0.439	0.611	1	−0.220
	Sig. (2-tailed)	0.000	0.000		0.000
VD; choroid	Pearson correlation^*∗*^	−0.243	−0.189	−0.220	1
	Sig. (2-tailed)	0.000	0.001	0.000	

VD: vessel density; SVC: superficial vascular complex; DVP: deep vascular complex; CC: choriocapillaris. ^*∗*^Statistically significant value (P-value <0.05).

## Data Availability

The datasets generated and/or analyzed in the current study are available from the corresponding author upon reasonable request.
